# Perivascular Adipose Tissue as an Indication, Contributor to, and Therapeutic Target for Atherosclerosis

**DOI:** 10.3389/fphys.2020.615503

**Published:** 2020-12-18

**Authors:** Yan Liu, Yan Sun, Chengping Hu, Jinxing Liu, Ang Gao, Hongya Han, Meng Chai, Jianwei Zhang, Yujie Zhou, Yingxin Zhao

**Affiliations:** Department of Cardiology, Beijing Anzhen Hospital, Capital Medical University, Beijing Institute of Heart Lung and Blood Vessel Disease, Beijing, China

**Keywords:** perivascular adipose tissue, atherosclerosis, inflammation, imaging, adipocytokines

## Abstract

Perivascular adipose tissue (PVAT) has been identified to have significant endocrine and paracrine functions, such as releasing bioactive adipokines, cytokines, and chemokines, rather than a non-physiological structural tissue. Considering the contiguity with the vascular wall, PVAT could play a crucial role in the pathogenic microenvironment of atherosclerosis. Growing clinical evidence has shown an association between PVAT and atherosclerosis. Moreover, based on computed tomography, the fat attenuation index of PVAT was verified as an indication of vulnerable atherosclerotic plaques. Under pathological conditions, such as obesity and diabetes, PVAT shows a proatherogenic phenotype by increasing the release of factors that induce endothelial dysfunction and inflammatory cell infiltration, thus contributing to atherosclerosis. Growing animal and human studies have investigated the mechanism of the above process, which has yet to be fully elucidated. Furthermore, traditional treatments for atherosclerosis have been proven to act on PVAT, and we found several studies focused on novel drugs that target PVAT for the prevention of atherosclerosis. Emerging as an indication, contributor to, and therapeutic target for atherosclerosis, PVAT warrants further investigation.

## Introduction

Atherosclerosis is a process in which the formation and buildup of lipid, cells, as well as matrix, and represents a major stage of atheromatous plaque formation. Atherosclerosis can cause myocardial infarction, stroke, and disabling peripheral artery disease, leading to high morbidity and mortality worldwide (Libby et al., 2019). The pathogenesis of atherosclerosis including endothelial dysfunction, inflammatory cells recruitment, vascular smooth muscle cells (VSMCs) proliferation and migration. During the process, cytokines are released by different types of cells and exert multiple effects, such as crosstalk among endothelial cells, vascular smooth muscle cells, inflammatory cells, and adipocytes (Tousoulis et al., 2016). The dynamic microenvironment plays a fundamental role in the pathogenesis of atherosclerosis.

The perivascular adipose tissue (PVAT) has long been considered a non-physiological structural tissue, but in the last decade, an increasing number of studies have identified it to have significant endocrine and paracrine functions, including the release of bioactive adipokines, cytokines, and chemokines (Szasz and Webb, 2012). Given the anatomical proximity between PVAT and the vascular wall, PVAT could play a crucial role in the pathogenic processes of atherosclerosis, mainly toward endothelial dysfunction (Virdis, 2016) and inflammatory cell recruitment (Omar et al., 2014). However, the pathophysiological characteristics of PVAT seem to be distinct in different anatomical locations (Gil-Ortega et al., 2015) and metabolic statuses (Molica et al., 2015; Ferrara et al., 2019), which remains to be clarified. In this review, we discuss the PVAT imaging features from the latest clinical evidence, the proatherogenic and antiatherogenic phenotypes of PVAT, the underlying molecular mechanisms and pathways, and potential therapeutic measures.

## Imaging Features of PVAT

### Ultrasound

Carotid intima-media thickness (IMT) is an index measure derived from high-resolution carotid ultrasound to assess the burden of atherosclerosis. As a complement, carotid extramedial thickness (EMT) focuses on the adventitial structure of arteries, which is mainly attributed to PVAT (Falk et al., 2009). Haberka et al. (2019) reported that EMT is an independent and strong predictor of significant internal carotid artery stenosis instead of obesity measurements. In addition, the combination of ultrasound indexes related to PVAT and the vascular wall was associated with more complex atherosclerotic coronary artery disease (CAD) in patients with high risk (Haberka et al., 2018). Carotid EMT is a promising index for early assessment of the clinical risk of atherosclerosis (Skilton, 2019). Similarly, intravascular photoacoustic ultrasound detected increased iliac arteries-PVAT in Ossabaw swine with metabolic syndrome, which was verified by histology as early-stage atherosclerotic changes (Kole et al., 2019).

### Computed Tomography

CT-based volumetric quantification of PVAT is feasible and highly reproducible (Schlett et al., 2009). Recent evidence has demonstrated that coronary artery-PVAT transforms from the lipid to aqueous phase during vascular inflammation, resulting in increased CT attenuation around the inflamed coronary artery (Antonopoulos et al., 2017). A novel non-invasive biomarker, the fat attenuation index (FAI), was designed to predict vulnerable atherosclerotic plaques and cardiac mortality (Antonopoulos et al., 2017; Antoniades et al., 2019; Lin et al., 2019). Moreover, the FAI was validated in a clinical cohort to show significant reduction around the culprit lesion; by contrast, there was no change around the stable atherosclerotic plaques (Antonopoulos et al., 2017). In CT imaging, we can identify adipose tissue with voxels between -190 and -30HU, and perform quantitative evaluation by calculating FAI or volume of PVAT, which helps the prediction of atherosclerosis.

### Magnetic Resonance Imaging

Due to its high cost and time consumption, studies based on MRI are limited and controversial. Randrianarisoa et al. (2018) showed a correlation between brachial artery PVAT and insulin resistance, while aorta PVAT is associated with carotid IMT, indicating that brachial artery PVAT and aorta PVAT may act differently as possible modulators of insulin resistance and subclinical atherosclerosis. However, Alkhalil et al. (2018) demonstrated the dissociation between the spatial distribution of PVAT and arterial wall thickening in the aorta and carotid arteries, which does not support that PVAT promotes atherosclerotic plaque through the paracrine route. The quantitative evaluations were implemented with different special software. Further research focusing on the assessment of PVAT with MRI is warranted.

### PVAT and Vascular Calcification

Vascular calcification was found to be associated with PVAT as a surrogate measure of atherosclerosis. The coronary artery segment covered by a myocardial bridge (MB) was isolated from the influence of PVAT. Verhagen et al. (2013) revealed that segments without an MB have a higher calcium score than segments covered with an MB. The association between calcium scores and MBs was influenced by local PVAT thickness (Verhagen et al., 2013). Moreover, women with systemic lupus erythematosus (SLE) were demonstrated to have greater aortic PVAT, which correlated with the calcification of different vascular beds (Shields et al., 2013). The summary of clinical imaging studies of PVAT and atherosclerosis is presented in Table 1. Based on above studies, it can be concluded that the increased volume or thickness of PVAT is associated with atherosclerosis. The enlargement of PVAT may indicates the transition from anti-atherogenic to pro-atherogenic phenotypes, which mediating the progression of atherosclerosis. The imaging features and pathological characteristics of PVAT needs further investigations.

**TABLE 1:** Overview of clinical imaging studies of perivascular adipose tissue (PVAT) and atherosclerosis.

Authors	Study design	Number of subjects	Imaging technology	Imaging index	Main findings
Haberka et al., 2019	Cross-sectional	391	Ultrasound	Carotid EMT	Carotid EMT was an independent and strong predictor of significant internal carotid artery stenosis
Haberka et al., 2018	Cross-sectional	215	Ultrasound	PATIMA index	The PATIMA index was associated with more complex CAD in high- and very-high-risk patients
Antonopoulos et al., 2017	Cross-sectional	273	CT	FAI	The FAI gradient around coronary arteries identified early subclinical coronary artery disease and vulnerable atherosclerotic plaques
Randrianarisoa et al., 2018	Cross-sectional	95	MRI	Volume	Brachial artery-PVAT was associated with insulin sensitivity, while aorta-PVAT was associated with carotid IMT
Alkhalil et al., 2018	Cross-sectional	29	MRI	Thickness	The spatial distribution of PVAT was uncorrelated with arterial wall thickening in the aorta and carotid arteries
Verhagen et al., 2013	Cross-sectional	128	CT	Thickness	The coronary artery segments without an MB have a higher calcium score than segments covered with an MB. The association between calcium scores and MBs was influenced by local PVAT thickness
Shields et al., 2013	Cross-sectional	135	CT	Volume	Women with SLE had greater aortic-PVAT, which correlated with calcification of different vascular beds

*EMT, extramedial thickness; PATIMA, periarterial adipose tissue intima media adventitia; CT, computed tomography; FAI, fat attenuation index; MRI, magnetic resonance imaging; IMT, intima-media thickness; MB, myocardial bridge; SLE, systemic lupus erythematosus.*

## Regional and Sex Differences in PVAT

### Regional Differences in PVAT

Several rodents and human studies found regional differences in PVAT impacting vascular function. Mouse abdominal aorta-PVAT is more inflamed than thoracic aorta-PVAT, which is independent of aging (Padilla et al., 2013). The phenotypic differences in PVAT between the regions of the aorta may account for the evidence that the abdominal aorta is more vulnerable to atherosclerosis than the thoracic aorta (Padilla et al., 2013; Victorio et al., 2016). A study based on multimodal non-linear optical imaging reported that mouse thoracic PVAT changes differentially from the initial stages to advanced stages of atherosclerosis and undergoes spatial impairment focused on atherosclerotic plaques (Kim et al., 2019). In humans, the phenotype of internal thoracic artery (ITA)-PVAT is closer to that of subcutaneous adipose tissue than that of coronary artery (CA)-PVAT (Numaguchi et al., 2019). ITA-PVAT appears to be protected from inflammation and consecutive adipose tissue remodeling, which may explain the decreased atherosclerotic plaque burden in the ITA (Numaguchi et al., 2019). Similarly, in patients with CAD, the genes related to inflammation, lipid metabolism and myocardial processes are differentially expressed in CA-PVAT and ITA-PVAT, demonstrating that PVAT is the key determinant in the development of atherosclerosis (Lu et al., 2017). In a community-based sample, individuals with high thoracic aorta-PVAT in the absence of high visceral adipose tissue were characterized by adverse cardiometabolic profiles, such as smoking and reduced high-density lipoprotein cholesterol (Britton et al., 2012). Therefore, compared with thoracic aorta and ITA, the vulnerability of abdominal aorta and CA to atherosclerosis may be explained by the regional differences of PVAT.

### Sex Differences in PVAT

Abdominal aortic aneurysm is a fatal disease with significant sexual dimorphism. Testosterone was verified to increase aneurysm rupture rates in female, which suggest the aortic vascular biology is regulated by sex chromosome (Alsiraj et al., 2017). Similarly, male is a risk factor of CAD, and estrogen could protect vascular from atherosclerosis. El Khoudary et al. (2015) demonstrated women gain cardiovascular fat after menopause, and the aortic PVAT volume is positively associated with estradiol reduction. Therefore, sex and sex hormones could play an important role in PVAT-mediated vascular atherosclerosis. Thromboxane and PGF2α mediate PVAT-induced vasoconstriction of porcine coronary artery in male and female pigs, respectively (Ahmad et al., 2017b). PVAT from female pigs inhibit contraction of porcine coronary artery, not male pigs (Ahmad et al., 2017a). Sex differences in PVAT have been reviewed in detail recently (Victorio et al., 2020), while the sex differences in PVAT-mediated atherosclerosis remains to be verified.

### Inflammation and Immunity of PVAT

Genome-wide expression analyses identified that the genes associated with the regulation of inflammation, angiogenesis, blood clotting, and vascular morphology were differentially expressed in human perivascular adipocytes and subcutaneous adipocytes (Chatterjee et al., 2013). Perivascular adipocytes signal to both inflammatory cells and endothelial cells, thus significantly modulating vascular inflammatory crosstalk in atherogenesis (Chatterjee et al., 2013).

### Proinflammatory and Anti-inflammatory Phenotypes of PVAT

Under physiological conditions, PVAT protects the artery against atherosclerosis by counteracting inflammation. Ren et al. (2019) founded that PVAT was formed around the disturbed blood flow-induced carotid atherosclerosis, and transplantation of thoracic PVAT from wild-type mice, which showed lower messenger RNA (mRNA) levels of inflammatory cytokines than thoracic PVAT from *ApoE*^–/–^ mice, decreased plaque macrophage content. Similarly, wild-type mouse periaortic PVAT transplantation inhibited atherosclerosis development by exerting TGF (transforming growth factor)-β1-mediated anti-inflammatory activity, which might involve M2 macrophages (Terada et al., 2018). Under pathological conditions, PVAT contributes to the formation process of atherosclerosis. Transplantation of abdominal aortic PVAT from high-fat diet (HFD)-fed mice increased aortic atherosclerosis and inhibited endothelium-dependent relaxation (Horimatsu et al., 2018). In the transplanted abdominal aortic PVAT tissue, MCP-1 (monocyte chemoattractant protein-1) and TNF (tumor necrosis factor)-α expression was elevated, while adiponectin expression was reduced (Horimatsu et al., 2018). Moreover, compared with PVAT surrounding a normal vessel (internal mammary artery), human PVAT of atherosclerotic arteries (coronary artery) showed more extensive inflammation, lymphangiogenesis, and fibrosis, probably due to local VEGF (vascular endothelial growth factor)-C, VEGF-D, and angiopoietin-2 overexpression (Drosos et al., 2019).

Similarly, the proinflammatory state of PVAT was identified with cardiovascular risk factors, such as hypertension, diabetes mellitus and peripheral arterial disease. In a hypertensive mouse model, T cells preferentially accumulated in aortic PVAT and could be increased by angiotensin II (Guzik et al., 2007). Accordingly, aortic PVAT-specific renin-angiotensin system activation contributes to accelerating atherosclerotic development in uninephrectomized mice (Kawahito et al., 2013). Angiotensin II type 1 receptor (AT1) plays an important role in the HFD-induced phenotypic alteration of aortic PVAT in *ApoE*^–/–^ mice, identified as higher expression of proinflammatory cytokines and inflammatory cell infiltration, and modulation of AT1 may exert beneficial effects on atherosclerosis (Irie et al., 2015). In patients with diabetes, carotid PVAT surrounding the atheromatous plaques showed an increase in the mRNA levels of TNF-a, MCP-1, and IL (interleukin)-6 (Hamlat-Khennaf et al., 2017). Patients with peripheral arterial disease showed higher gene expression of TNF-α in renal artery-PVAT (Cejkova et al., 2017). According to Wakana et al. (2015), maternal HFD accelerated atherosclerosis development in offspring, as the increasing thoracic aorta-PVAT specific inflammatory response mediated by higher expression of macrophage colony-stimulating factor. In contrast, an Ossabaw pig experiment exhibited disconnection between left anterior descending coronary artery-PVAT inflammation and HFD-induced cardiometabolic dysfunction (Vieira-Potter et al., 2015).

### Inflammatory Cells of PVAT

Human aortic PVAT produces different chemokines, such as IL-8 and MCP-1, which induce the chemotaxis of granulocytes, monocytes, and activated T cells (Henrichot et al., 2005). In patients with CAD, the ratio of macrophages was significantly higher in the coronary arterial wall, and a close relationship was identified between the ratio of macrophages in the arterial wall and PVAT (Verhagen et al., 2012; Kralova Lesna et al., 2015). Coronary PVAT macrophages were associated with stenosis of the adjacent vessel, and M2 macrophages were more abundant than M1 macrophages in PVAT (Verhagen et al., 2014). The inflammatory cells in PVAT seem to play a crucial role in atherogenesis. A postmortem study revealed that the plaque/media ratio increased with the area and macrophages of coronary PVAT (Verhagen et al., 2012). The area of coronary PVAT was related to the presence of a lipid core and the infiltration of macrophages and lymphocytes in atherosclerotic plaques (Verhagen et al., 2012). Similarly, Farias-Itao et al. (2019) identified the correlation between inflammatory cells in coronary PVAT and atherosclerotic plaque features. In periplaque coronary PVAT, the density of CD68^+^ macrophages and CD20^+^ B lymphocytes increased with the size of the plaque, and coronary PVAT surrounding unstable atherosclerotic plaques exhibited greater CD68^+^ macrophages than surrounding stable lesions (Farias-Itao et al., 2019). In addition, in the development of atherogenesis, commensal microbes activate B2 cells in PVAT through lipid metabolism-independent mechanisms (Chen et al., 2016). B1 cells in PVAT were found to produce immunoglobulin type M antibodies to limit atherosclerosis development (Srikakulapu et al., 2017).

### PVAT and Immunity

Both rodents and human experiments suggested that PVAT produced complement proteins C3 and C4, which bind to elastin fibers and collagen within the adventitia, leading to increased vascular atherosclerosis and vascular calcification (Shields et al., 2011; Nagaraj et al., 2015). In rats, immunization intracutaneously with native human (nh)-LDL or nh-HDL induced increased PVAT volume and atherosclerosis-like changes in the aortic wall: leukocytes accumulation, intima destruction and media structure disruption (Fomina et al., 2017). Therefore, the immune response toward native lipoproteins might be adipogenic and atherogenic.

Taken together, the inflammation and immunity of PVAT are key determinants in the pathogenesis of atherosclerosis, which deserve further exploration.

### Endothelial Function and PVAT-Derived Adipocytokines

Endothelial function is largely based on endothelial nitric oxide synthase (eNOS) function and activity (Daiber et al., 2019). PVAT may regulate arterial tone by releasing diffusible vasorelaxation factors, which, through endothelium-derived NO production, compensate for impaired vasodilatation in metabolic disorders (Kagota et al., 2017; Baltieri et al., 2018). On the other hand, factors released from PVAT may inhibit endothelial NO production and induce vasocontraction by increasing the expression of Cav-1 protein (Lee et al., 2014). AMP-activated protein kinase (AMPK) was revealed to regulate adipocyte metabolism, adipose biology and vascular function (Almabrouk et al., 2014). Activation of AMPK restrains the production of PVAT-released adipokines and prevents endothelial dysfunction by increasing the bioavailability of NO (Gao et al., 2018). Compared with the PVAT of the thoracic aorta, eNOS-derived NO production decreased in the PVAT of the abdominal aorta, suggesting the susceptibility of the abdominal aorta to vascular injury (Victorio et al., 2016). It is now well accepted that adiponectin, as a protective adipocytokine secreted by PVAT, possesses anti-inflammatory, insulin-sensitizing, and vasodilating properties (Milutinovic et al., 2019). In HFD mice, endothelium-mediated vascular relaxation was impaired, and subcutaneously pumped adiponectin was demonstrated to increase eNOS phosphorylation and decrease PVAT inflammation, thereby restoring endothelial cell function (Sena et al., 2017). Consistently, endothelium-dependent relaxation was reduced in obese patients (Virdis, 2016; Cybularz et al., 2017). Endogenous adiponectin expression was increased in PVAT to maintain endothelial function (Cybularz et al., 2017). Moreover, in patients with atherosclerosis, peroxidation products produced in the vascular wall promote adiponectin gene expression in PVAT through a peroxisome proliferator-activated receptor-γ (PPARγ)-dependent mechanism (Margaritis et al., 2013). Then, adiponectin restores eNOS coupling to improve the redox state (Margaritis et al., 2013). In addition, PPARγ deficiency in PVAT enhances atherosclerosis and results in vascular and systemic inflammation (Xiong et al., 2018). Additionally, PVAT-secreted adiponectin promotes macrophage autophagy by suppressing the Akt/FOXO3a signaling pathway, subsequently suppressing plaque formation (Li et al., 2015). Growing studies focus on glucose metabolism of endothelial and VSMCs. Diabetes restrains glycolysis upregulation in endothelial cells under hypoxia conditions (Primer et al., 2020). High concentration of glucose promotes P. gingivalis invasion of human aortic SMCs, then initiates osteogenic phenotype switch, which results in calcification (Chen et al., 2018). Adiponectin also participates in the regulation of related process. High glucose leads to vascular adiponectin resistance, and contributes to diabetic endothelial dysfunction (Liu et al., 2015). High glucose could reduce adiponectin expression of stromal cells in epicardial adipose tissue, which induce an inflammatory paracrine process in endothelial cells (Fernández-Trasancos et al., 2016). Besides, adiponectin could reduce VSMCs proliferation and migration which induced by high glucose (Cersosimo et al., 2020). Leptin was perceived as a risk factor in atherosclerosis progression, which promote endothelial dysfunction, PVAT inflammation and vascular smooth muscle cell phenotypic switching (Dib et al., 2014; Li et al., 2014; Payne et al., 2014). Circulating plasma leptin negatively correlates with endothelial dependent vasodilation (Gonzalez et al., 2013). In Ossabaw with metabolic syndrome, the increased epicardial PVAT leptin was revealed to exacerbate coronary endothelial dysfunction via a protein kinase C-β-dependent pathway (Payne et al., 2010). In female mice, leptin was found to induce endothelial dysfunction through aldosterone-dependent mechanisms (Huby et al., 2016). In addition, patients with coronary artery disease were identified to have more severe hypoxia in local tissue and higher expression of leptin in PVAT, as well as increased inflammation, vascularization, and fibrosis, accounting for the increased atherosclerotic plaque burden within the coronary arteries (Drosos et al., 2016). A study of ewes showed that leptin increased IL-1B and TNF-a gene expression, which may moderate the inflammatory reaction progress in PVAT (Krawczynska et al., 2019).

Perivascular adipose tissue dysfunction is characterized by its inflammatory state, reduced production of vasoprotective adipocyte-derived relaxing factors such as adiponectin and omentin (Van de Voorde et al., 2013) and augmented production of proinflammatory factors such as leptin, resistin, cytokines (TNF-α and IL-6) and chemokines (MCP-1 and RANTES) (Nosalski and Guzik, 2017). These factors promote inflammatory cell infiltration and induce eNOS dysfunction, ultimately leading to the development of atherosclerotic diseases (Van de Voorde et al., 2013; Nosalski and Guzik, 2017; Aroor et al., 2018).

### PVAT and Thermoregulation

Perivascular adipose tissue is similar to brown adipose tissue and is essential for intravascular thermoregulation upon cold acclimation, which plays a protective role in the pathogenesis of atherosclerosis (Chang et al., 2012). Proteomics analysis of PVAT from mice identified that cold exposure improves endothelial function and inhibits atherosclerosis (Chang et al., 2012). Moreover, an experiment in ferrets found that cold exposure downregulated gene expression in aortic PVAT, which was associated with the immune response, cell cycle and gene expression regulation (Reynes et al., 2017). Aortic PVAT exhibited an anti-inflammatory response to cold acclimation and demonstrated a protective effect against atherogenesis (Reynes et al., 2017). Different proteins mediated the thermoregulatory response of PVAT. As a mitochondrial outer membrane protein, the CDGSH iron sulfur domain 1 protein (referred to as mitoNEET) could regulate oxidative capacity and the browning of adipose tissue (Xiong et al., 2017). MitoNEET in PVAT contributes to PVAT-dependent thermogenesis, thereby preventing atherosclerosis development (Xiong et al., 2017). In addition, Tang et al. (2018) demonstrated ribosomal protein S3A as a key factor in modulating the brown fat-specific gene uncoupling protein 1 and carbon metabolic enzymes in epicardial adipose tissue to prevent CAD. These findings suggested that the thermogenic capacity of PVAT is beneficial to the protection of atherosclerosis.

### Proatherogenesis-Related Molecular Mechanisms and Pathways

With respect to the proatherogenic role of PVAT, different proteins, bioactive factors and pathways have been investigated. Coronary PVAT of Ossabaw swine was identified to attenuate vasodilation through the inhibitions of vascular smooth muscle K + channels (Noblet et al., 2015). Carotid PVAT transplantation leads to endothelial dysfunction and accelerated atherosclerosis in *ApoE*^–/–^ mice, and it could be blocked by neutralization of Psgl-1 (P-selectin glycoprotein ligand-1) (Ohman et al., 2011). Consistently, Psgl-1 deficiency was revealed to prevent mesenteric PVAT inflammation and endothelial dysfunction in obese mice (Wang et al., 2012). Thus, Psgl-1 may play a crucial role in the formation of atherosclerotic lesions. Moreover, in HFD-fed rabbits, especially in carotid PVAT(+) groups, CRP (C-reactive protein) significantly promoted endothelial dysfunction, which might be mediated by activating the inflammatory response of adipose tissue (Chen et al., 2013). CRP was also found to contribute to vasa vasorum growth by activating the proangiogenic activity of adipose-derived stem cells (Chen et al., 2016), which promote the pathogenesis of atherosclerosis.

Xpr1 and Taf3, a regulator of macrophage differentiation and a core transcription factor, which were detected in mouse PVAT, were significantly upregulated during atherosclerosis, suggesting a role in the pathogenesis of atherosclerosis (Hueso et al., 2016). Interestingly, hepatokine fibroblast growth factor 21 exerts secretome-modulating responses in human perivascular adipocytes, establishing a novel liver-PVAT-blood vessel axis that possibly accounts for vascular inflammation and atherosclerosis (Berti et al., 2016). Moreover, miR-19b in endothelial cell-derived microvesicles promotes atherosclerosis progression by increasing PVAT-specific inflammation by diminishing SOCS3 (suppressor of cytokine signaling 3) expression (Li et al., 2018). In addition, endoplasmic reticulum stress in PVAT destabilizes atherosclerotic plaques by increasing GM-CSF (granulocyte macrophage colony stimulating factor) in a paracrine manner via the transcription factor NF-κB (Ying et al., 2018). In addition, higher levels of inhibiting protein of activated STAT1 (PIAS1) and diminished expression of NF-κB- or STAT1-regulated genes involved in adipocyte inflammation, differentiation, senescence and apoptosis were present in mouse PVAT, while PIAS1 was decreased in the PVAT of patients with atherosclerosis (Schutz et al., 2019), suggesting that STAT1- or NF-κB-regulated genes may mediate the proatherogenesis effects of PVAT. Taken together, PVAT may contribute to atherosclerosis through promoting endothelial dysfunction, accelerating inflammation reaction, and motivating vasa vasorum growth. The underlining molecular mechanism warrants further clarification.

## Therapeutic Targets

### Smoking Cessation

Nicotine, as the key active component of cigarette smoke, was proven to correlate with PVAT inflammation and contribute to endothelial dysfunction. The PVAT of smokers showed higher expression and activity of the P2 × 7 receptor-inflammasome complex, which contributed to the proinflammatory status (Rossi et al., 2014). Nicotine induced mature adipocyte dysfunction, thus leading to the abnormal secretion of adiponectin and inflammatory adipokines and exacerbating endothelial inflammation (Wang et al., 2016). In addition, adipocytes promote nicotine-induced apoptosis of endothelial cells through the NF-κB pathway (Liu et al., 2017). Smoking cessation could prevent vascular atherosclerosis induced by PVAT dysfunction.

### Antidiabetic Drugs

Pioglitazone, an insulin sensitizer, alleviated PVAT oxidative damage induced by fructose treatment and prominently diminished proinflammatory markers in *ApoE*^–/–^ mice (Quesada et al., 2018). Additionally, pioglitazone dramatically downregulated the expression of vascular cell adhesion molecule-1 (VCAM-1) and matrix metalloprotein-9 (MMP-9), as well as the activity of MMP-9 in the aortic media wall, and markedly decreased the accumulation of macrophages and lipids in atheroma plaques (Quesada et al., 2018). Pioglitazone treatment increased adiponectin serum level (Negrotto et al., 2016), which enhance endothelial mediated vasodilation (Quinn et al., 2010) and increase the number and function of endothelial progenitor cells (Werner et al., 2007), thereby improve vascular dysfunction (Fernandez et al., 2008).

It was reported that glucagon-like peptide-1 (GLP-1)-based therapeutic methods may positively affect autophagy in PVAT, thus improving endothelial dysfunction caused by obesity (Costantino and Paneni, 2019). Both dipeptidyl peptidase-4 (DPP-4) inhibitors and GLP-1 receptor agonists are modulators of this process. In addition, PVAT is a source of DPP-4, and its biology could be influenced by DPP-4 inhibition (Akoumianakis and Antoniades, 2017). DPP-4 inhibition was associated with diminished oxidative stress and local inflammation both in the PVAT and the vascular wall, potentially regulating atherogenesis progression *in vivo* (Akoumianakis and Antoniades, 2017). For instance, teneligliptin could decrease the expression of a major NADPH oxidase subunit, Nox-4, and a macrophage marker in perivascular adipocytes of normoglycemic *ApoE*^–/–^ mice (Salim et al., 2017).

Sodium glucose cotransporter 2 (SGLT2) inhibitors were revealed to suppress the inflammation of PVAT and attenuate atherogenesis in an *in vivo* mouse model. Empagliflozin ameliorated the RNA expression of inflammatory factors in PVAT, attenuated diabetes-induced endothelial dysfunction, and decreased the atherosclerotic lesion area in the aortic arch of diabetic *ApoE*^–/–^ mice (Ganbaatar et al., 2020). Empagliflozin suppressed the expression of PDGF-B in PVAT macrophages, thereby attenuating neointimal hyperplasia after wire injury in HFD-fed mice (Mori et al., 2019).

### Other Drugs

Short-term intensive atorvastatin therapy attenuates PVAT inflammation by inhibiting the 5-lipoxygenase pathway, thus significantly ameliorating endothelial dysfunction in HFD rabbits (Wang et al., 2014). The selective Mas receptor agonist AVE0991 (angiotensin 1-7 mimetic) affected macrophage differentiation and recruitment into the perivascular space, exhibiting anti-inflammatory and antiatherosclerotic actions during the early stages of atherosclerosis in *ApoE*^–/–^ mice (Skiba et al., 2017).

Morinda citrifolia leaf extract was shown to reduce PVAT deposition while preventing atherosclerosis through lipid elimination and anti-inflammatory reactions in ovariectomized HFD-fed mice (Chong et al., 2018). Similarly, pseudoprotodioscin (PPD), a phytoestrogen isolated from Dioscorea nipponica Makino, alleviated atherosclerotic lesions and exerted estrogenic and anti-inflammatory properties in ovariectomized HFD-fed *ApoE*^–/–^ mice (Sun et al., 2020).

Polysaccharide peptide from Ganoderma lucidum, as one source of antioxidant, had a significant effect in decreasing H_2_O_2_ levels, PVAT thickness, the number of foam cells, and atherosclerotic plaque width in mice (Andri Wihastuti et al., 2015). 10,12 Conjugated linoleic acid (10,12 CLA)-supplemented mice were characterized by enrichment of M2 macrophages in artery lesions and surrounding PVAT, which contributed to the antiatherosclerotic role of 10,12 CLA (Kanter et al., 2018). Dietary ethanolic extract of mangosteen pericarp significantly diminished the expression of VCAM-1 and decreased the thickness of PVAT and IMT in HFD-fed mice (Wihastuti et al., 2019).

Smoking, antidiabetic drugs and other drugs have been verified to act on PVAT, reducing inflammation reaction and endothelial dysfunction, thus preventing atherosclerosis. PVAT is emerging as the therapeutic target of atherosclerosis.

## Conclusion

**FIGURE 1:**
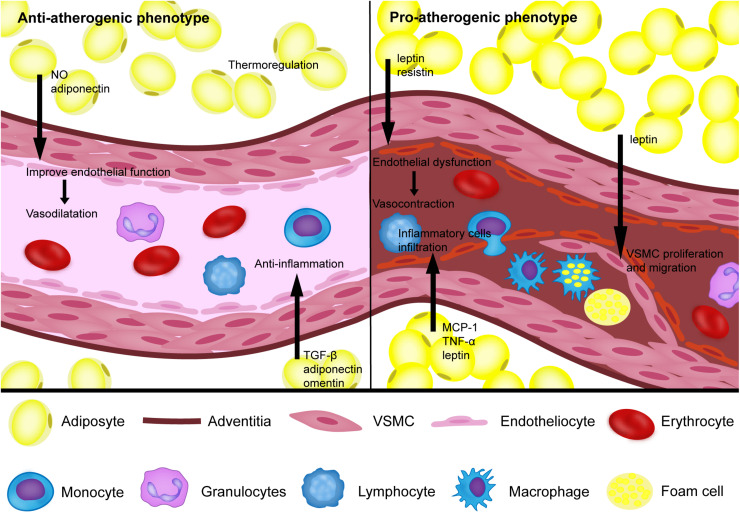
The proatherogenic and antiatherogenic phenotypes of perivascular adipose tissue. Under physiological conditions, perivascular adipose tissue (PVAT) can secrete NO, TGF-β, adiponectin, omentin, and other bioactive factors to reduce the inflammatory response and improve endothelial function, which helps vasodilation. In addition, PVAT also acts as an antiatherogenic phenotype through thermoregulation. Under pathological conditions, such as obesity and diabetes, PVAT shows a proatherogenic phenotype by increasing the release of MCP-1, TNF-a, leptin, resistin and other bioactive factors to induce endothelial dysfunction, inflammatory cell infiltration, vascular smooth cell (VSMC) proliferation and migration.

Growing clinical evidence based on imaging technology suggests that the phenotype of PVAT is associated with inflammation and the metabolic profile of the corresponding vasculature as the key determinants for the pathogenesis of atherosclerosis. Under physiological conditions, PVAT acts as an antiatherogenic phenotype by releasing vasodilating factors, exerting an anti-inflammatory response, and thermoregulation. Under pathological conditions, such as obesity and diabetes, PVAT shows the proatherogenic phenotype by increasing the release of adipocytokines and chemokines, which induce endothelial dysfunction, inflammatory cell infiltration, VSMC proliferation and migration, thus contributing to the augmented atherosclerotic plaque burden of the arteries (Figure 1). Improving cardiovascular risk stratification based on PVAT imaging could help identify individuals with unstable lesions. Furthermore, the traditional treatments for atherosclerosis, such as smoking cessation, atorvastatin and antidiabetic drugs, have been proven to act on PVAT. Rapid progress in pharmaceutical research has led to the identification of novel drugs targeting PVAT for the prevention of atherosclerosis. Defining the mechanism of PVAT regulation of vascular homeostasis warrants more and better controlled investigations.

## Author Contributions

YL and YXZ conceived the original scope of this manuscript. YL, YS, CH, JL, and AG wrote specific sections. HH, MC, JZ, YJZ, and YXZ critically reviewed and revised the final manuscript. All authors read and approved the final manuscript.

## Conflict of Interest

The authors declare that the research was conducted in the absence of any commercial or financial relationships that could be construed as a potential conflict of interest.

## Abbreviations

10,12 CLA: 10,12 conjugated linoleic acid
AMPK: AMP-activated protein kinase
AT1: angiotensin II type 1 receptor
CA: coronary artery
CAD: atherosclerotic coronary artery disease
CRP: C-reactive protein
CT: computed tomography
DPP-4: dipeptidyl peptidase-4
EMT: extramedial thickness
eNOS: endothelial nitric oxide synthase
FAI: fat attenuation index
GLP-1: glucagon like peptide-1
GM-CSF: granulocyte macrophage colony stimulating factor
HFD: high-fat diet
IL: interleukin
IMT: intima-media thickness
ITA: internal thoracic artery
MB: myocardial bridge
MCP-1: monocyte chemoattractant protein-1
mitoNEET: CDGSH iron sulfur domain 1 protein
MMP-9: matrix metalloprotein-9
MRI: magnetic resonance imaging
mRNA: messenger RNA
Nh: native human
PIAS1: inhibiting protein of activated STAT1
PPARγ: peroxisome proliferator-activated receptor-γ
PPD: pseudoprotodioscin
Psgl-1: P-selectin glycoprotein ligand-1
PVAT: perivascular adipose tissue
SGLT2: sodium glucose cotransporter 2
SLE: systemic lupus erythematosus
SOCS3: suppressor of cytokine signaling 3
TGF: transforming growth factor
TNF: tumor necrosis factor
VCAM-1: vascular cell adhesion molecule-1
VEGF: vascular endothelial growth factor
VSMCs: vascular smooth muscle cells.
